# Autophagic Signaling and Proteolytic Enzyme Activity in Cardiac and Skeletal Muscle of Spontaneously Hypertensive Rats following Chronic Aerobic Exercise

**DOI:** 10.1371/journal.pone.0119382

**Published:** 2015-03-23

**Authors:** Elliott M. McMillan, Marie-France Paré, Brittany L. Baechler, Drew A. Graham, James W. E. Rush, Joe Quadrilatero

**Affiliations:** Department of Kinesiology, University of Waterloo, Waterloo, Ontario, Canada; University of Rome La Sapienza, ITALY

## Abstract

Hypertension is a cardiovascular disease associated with deleterious effects in skeletal and cardiac muscle. Autophagy is a degradative process essential to muscle health. Acute exercise can alter autophagic signaling. Therefore, we aimed to characterize the effects of chronic endurance exercise on autophagy in skeletal and cardiac muscle of normotensive and hypertensive rats. Male Wistar Kyoto (WKY) and spontaneously hypertensive rats (SHR) were assigned to a sedentary condition or 6 weeks of treadmill running. White gastrocnemius (WG) of hypertensive rats had higher (p<0.05) caspase-3 and proteasome activity, as well as elevated calpain activity. In addition, skeletal muscle of hypertensive animals had elevated (p<0.05) ATG7 and LC3I protein, LAMP2 mRNA, and cathepsin activity, indicative of enhanced autophagic signaling. Interestingly, chronic exercise training increased (p<0.05) Beclin-1, LC3, and p62 mRNA as well as proteasome activity, but reduced (p<0.05) Beclin-1 and ATG7 protein, as well as decreased (p<0.05) caspase-3, calpain, and cathepsin activity. Left ventricle (LV) of hypertensive rats had reduced (p<0.05) AMPKα and LC3II protein, as well as elevated (p<0.05) p-AKT, p-p70S6K, LC3I and p62 protein, which collectively suggest reduced autophagic signaling. Exercise training had little effect on autophagy-related signaling factors in LV; however, exercise training increased (p<0.05) proteasome activity but reduced (p<0.05) caspase-3 and calpain activity. Our results suggest that autophagic signaling is altered in skeletal and cardiac muscle of hypertensive animals. Regular aerobic exercise can effectively alter the proteolytic environment in both cardiac and skeletal muscle, as well as influence several autophagy-related factors in skeletal muscle of normotensive and hypertensive rats.

## Introduction

Hypertension is a cardiovascular disease most commonly associated with pathology of blood vessels and heart, but also skeletal muscle. Underlying a number of skeletal and cardiac muscle pathologies are alterations to autophagic signaling and flux. Macroautophagy (herein referred to as autophagy) is a catabolic process responsible for the bulk and selective degradation of aggregated or damaged proteins and organelles [1]. In terminally differentiated cells such as skeletal and cardiac myocytes, basal autophagy is essential to prevent the accumulation of damaged/dysfunctional proteins and organelles which can be detrimental to the cell [2,3]; however, increased autophagy can contribute to pathological tissue remodeling [4,5]. Furthermore, modification of autophagic proteins such as Beclin-1 and ATG5 by apoptosis-related enzymes can promote cell death [6,7]. Collectively, the literature supports that a fine regulation of autophagy is important for proper tissue function and cellular homeostasis.

Upon induction of autophagy, two kinase complexes, ULK1/ATG13 and Beclin-1/Vps34 are critical in the initiation and nucleation of a phagophore (isolation membrane) [1]. A number of autophagy-related proteins (ATG3, ATG4, ATG7, ATG10) and two conjugation systems (ATG12-ATG5 and LC3-PE) allow for the elongation and closure of the phagophore to form a double-membrane vesicle known as the autophagosome [8,9]. In addition to its role in phagophore expansion, LC3 can selectively target specific proteins and organelles to the autophagosome via recognition of the ubiquitin-binding protein, p62 [10]. Following sequestration of the targeted proteins or organelles, the outer membrane of the autophagosome binds to the lysosome, a process involving LAMP2 proteins [1]. Lysosomal enzymes such as cathepsins then degrade the autophagic cargo, resulting in their release into the cytosol to be used by the cell [11].

Altering autophagy and autophagy-related proteins can have beneficial effects on tissue function and viability in a number of disease models. Acute exercise has been shown to alter the expression of autophagic proteins and potentially autophagic flux [12–15]. Limited data has also shown alterations in autophagy during exercise training [16,17]. It is possible that increasing autophagic flux through exercise may improve cellular processes and function by reducing the amount of toxic protein aggregates and dysfunctional organelles. Therefore, one of the mechanisms by which exercise training may lead to cellular benefits and decreased apoptosis in skeletal and cardiac muscle is by promoting autophagy. However, current evidence for the effects of exercise training on autophagy and autophagy-related protein and gene expression is limited.

Work from our lab has shown that skeletal muscle from hypertensive rats is under a state of increased apoptotic stress [18–20]. Interestingly, cardiac and skeletal muscle (particularly fast muscle) from hypertensive animals also show signs of altered autophagy [21]. Therefore, the purpose of this study was to further characterize the expression of autophagic signaling proteins and genes in both skeletal and cardiac muscle of hypertensive rats. In addition, we examined the effects of 6 weeks of endurance exercise on autophagic signaling and proteolytic enzyme activity in skeletal and cardiac muscle of normotensive and hypertensive rats.

## Materials and Methods

### Animals

Male Wistar Kyoto (WKY) and spontaneously hypertensive rats (SHR) were purchased from Harlan (Indianapolis, IN). Animals were group housed on a 12:12 hr reverse light dark cycle, and given access to standard rodent lab chow (Harlan) and tap water *ad libitum*. Morphological and blood pressure data for these animals has previously been published by our group, and demonstrate significantly elevated body mass normalized heart weight, body mass normalized LV mass, and blood pressure in SHR compared to WKY rats. Exercise training did not affect body mass, body mass normalized heart weight, or resting blood pressure [22]. There were no mortalities in any group throughout the study. All animal procedures were approved by the University of Waterloo Animal Care Committee (ACC).

### Exercise protocol

Rats were randomly assigned to an aerobic exercise training (EX) or sedentary (SED) condition, resulting in the following groups: WKYSED (n = 12), SHRSED (n = 12), WKYEX (n = 12), and SHREX (n = 12). At eleven weeks of age, rats were acclimatized to a motorized treadmill (Wood’s Chambersburg, PA, USA) with a single 10 min bout of running (5–10 m/min at a 0% grade). Exercise consisted of a progressive aerobic treadmill running protocol (6 wks, 5 days/wk) that reached a final intensity and duration of 21 m/min, 4.5% grade, 45 min/day by the end of week 3. For the SED condition, animals were restricted to cage-bound activity for 6 weeks and exposed to treadmill noise and vibration on the same number of occasions and same duration as EX animals. A significant training response in these animals was confirmed through increased citrate synthase and βHAD enzyme activity in WG, as well as increased content of several mitochondrial proteins in soleus [20,22].

### Immunoblotting

Immunoblot analysis was performed as previously described [20,21,23]. The following primary antibodies were used: AMPKα (Cat. #2532), p-AMPKα (Thr^172^) (Cat. #2535), AKT (Cat. #9272), p-AKT (Thr^308^) (Cat. #9275), FoxO3a (Cat. #9467), p-FoxO3a (Ser^318/321^) (Cat. #9465), ULK1 (Cat. #8054), p-ULK1 (Ser^467^) (Cat. #4634), p-ULK1 (Ser^555^) (Cat. #5869), ATG12-5 (Cat. #4180), ATG4B (Cat. #5299), ATG7 (Cat. #8558), Beclin-1 (Cat. #3738), LC3 (Cat. #2775), p70S6K (Cat. # 2708), p-p70S6K (Thr^421^/Ser^424^) (Cat. # 9204) (all from Cell Signaling; Danvers, MA, USA), MAFbx (sc-33782), MuRF1 (Cat. #sc-32920), Bcl-xL (Cat. #sc-8392), Bcl-2 (Cat. #sc-7382), p-Bcl-2 (Ser^87^) (Cat. #sc-16323) (all from Santa Cruz Biotechnology; Dallas, TX, USA), BNIP3 (Cat. #B7931) (Sigma-Aldrich, St. Louis, MO, USA), and p62 (Cat. #GP62-C) (Progen, Heidelberg, Germany). Antibodies were diluted to concentrations ranging from 1:100 to 1:1000, based on dilution curve optimization tests. Following primary antibody incubation, membranes were washed with TBS-T, incubated with appropriate horseradish peroxidase (HRP)-conjugated secondary antibodies (Santa Cruz Biotechnology) for 1 hr at room temperature, washed with TBS-T, and visualized using enhanced chemiluminescence (ECL) Western blotting detection reagents (GE Healthcare, Little Chalfont, UK) and the ChemiGenius 2 Bio-Imaging System (Syngene, Cambridge, UK). Precision Plus Protein WesternC Standards and Precision Protein Strep-Tactin HRP Conjugate (Bio- Rad Laboratories) were used to estimate the molecular weight for each protein. Ponceau S (Sigma-Aldrich) staining of membranes was used to confirm equal protein loading and quality of transfer.

### Quantitative RT-PCR

Muscle was homogenized in ice-cold Trizol Reagent (Sigma-Aldrich) with 0.2 mg mL^-1^ glycogen. RNA was isolated by chloroform phase separation and alcohol precipitation, assessed for integrity by agarose gel electrophoresis, and its concentration determined using the NanoDrop 2000 spectrophotometer (Thermo Scientific, Maltham, MA, USA). cDNA synthesis was performed by reverse transcription using the qScript cDNA Synthesis Kit (Quanta Biosciences, Gaithersburg, MA, USA) [21]. PCR primers for cDNA amplification were assessed using Oligoanalyzer (Integrated DNA Technologies, Coralville, IA, USA) and GeneBlast (NCBI, Bethesda, MD, USA), and constructed (Sigma-Aldrich) as follows: Beclin-1 forward: 5′-ACAGCTCCATTACTTACCACAGCCC-3′, reverse: 5′-AATCTTCGAGAGACACCATCCTGGC-3′; LAMP2 forward: 5′-AGCAGGTGGTTTCCGTGTCTC-3′, reverse: 5′-GGAAGTTGTCTTCATCTGCACTGCA-3′; LC3 forward: 5′-CGAGAGCGAGAGAGATGAAGACGG-3′, reverse: 5′-GGTAACGTCCCTTTTTGCCTTGGTA-3′; p62 forward: 5′-AGAATGTGGGGGAGAGCGTGGC-3′, reverse: 5′-GGGTGTCAGGCGGCTTCTCTT-3′; and β-actin forward: 5′-CTGGCTCCTAGCACCATGAAGAT-3′, reverse: 5′-GGTGGACAGTGAGGCCAGGAT-3′. PCR cDNA amplification was performed with the PerfeCTa SYBR Green SuperMix Low ROX Kit (Quanta Biosciences), and analyzed with the 7500 RT-PCR System (Applied Biosystems, Invitrogen Life Technologies, Carlsbad, CA, USA). Expression was normalized to levels of β-actin mRNA.

### Proteolytic and proteasomal enzyme activity

To assess cathepsin activity, muscle was homogenized in ice-cold lysis buffer (without protease inhibitors), centrifuged at 1,000 *g* for 10 min at 4°C, and the supernatant collected. Supernatant was incubated (in duplicate) in the dark at 30°C in reaction buffer (50 mM sodium acetate, 8 mM DTT, 4 mM EDTA, 1 mM Pefabloc; pH 5.0) with 50 μM of the fluorogenic substrate z-FR-AFC (Enzo Life Sciences, Farmingdale, NY, USA) [21]. Fluorescence was determined using a SPECTRAmax Gemini XS microplate spectrofluorometer (Molecular Devices, Sunnyvale, CA, USA) with excitation and emission wavelengths of 400 nm and 505 nm, respectively, for 30 min at 30°C. Cathepsin activity was normalized to total protein concentration, and expressed as fluorescence intensity in arbitrary units (AU) per mg protein.

To assess the enzymatic activity of caspase-3, muscle was homogenized in ice-cold lysis buffer without protease inhibitors. Samples were centrifuged for 10 min at 1,000 g at 4°C, the supernatant collected, and incubated (in duplicate) in the dark with the fluorogenic substrate Ac-DEVD-AMC (Enzo Life Sciences) [20]. Fluorescence was measured using a SPECTRAmax Gemini XS microplate spectrofluorometer (Molecular Devices) with excitation and emission wavelengths of 360 nm and 440 nm, respectively. Caspase activity was normalized to total protein content, and expressed as fluorescence intensity in AU per mg protein.

Calpain activity was assessed using the fluorogenic substrate Suc-LLVY-AMC (Enzo Life Sciences). Supernatant was incubated in the dark with substrate, or substrate and the calpain inhibitor Z-LL-CHO (Enzo Life Sciences) at 37°C. Fluorescence was measured using a SPECTRAmax Gemini XS microplate spectrofluorometer (Molecular Devices) with excitation and emission wavelengths of 380 nm and 460 nm, respectively. Calpain activity was calculated as the difference in fluorescence from homogenate incubated with and without the inhibitor [24], normalized to total protein content, and expressed as fluorescence intensity in AU per mg protein.

Chymotrypsin-like activity of the proteasome was assessed using the fluorogenic substrate Suc-LLVY-AMC (Enzo Life Sciences) [25]. Supernatant was incubated in the dark with substrate, or substrate and the proteasome inhibitor epoxomicin (Cayman Chemical; Ann Arbor, MI, USA) at 30°C. Fluorescence was measured using a SPECTRAmax Gemini XS microplate spectrofluorometer (Molecular Devices) with excitation and emission wavelengths of 380 nm and 460 nm, respectively. Proteasome activity was calculated as the difference in fluorescence from homogenate incubated with and without the inhibitor, normalized to total protein content, and expressed as fluorescence intensity in AU per mg protein.

### Measurement of reactive oxygen species generation

Muscle reactive oxygen species (ROS) generation was determined using the fluorogenic substrate DCFH-DA as previously described [20,24]. Briefly, muscle was homogenized in ice-cold buffer (250 mM sucrose, 20 mM HEPES, 10 mM KCl, 1 mM EDTA, 1 mM EGTA, 1 mM DTT; pH 7.4) with protease inhibitors (Complete Cocktail; Roche Diagnostics). Muscle homogenate (in duplicate) was incubated in the dark at 37°C with 5 μM DCFH-DA (Invitrogen). DCF fluorescence was determined using a SPECTRAmax Gemini XS microplate spectrofluorometer (Molecular Devices) with excitation and emission wavelengths of 490 nm and 525 nm, respectively. Fluorescence was normalized to total protein content, and expressed as AU per mg of protein

### Statistical analysis

All data were analysed using a 2 (WKY vs. SHR) x 2 (SED vs. EX) factorial ANOVA. Tukey’s post-hoc test was used where appropriate. In a single instance (WG calpain activity) a Student’s T-test was used to analyse differences between the SHRSED and SHREX groups. Statistical significance was considered at p<0.05.

## Results

### Proteolytic enzyme activity, ROS generation, and ubiquitin-proteasome system (UPS)

**Fig 1:**
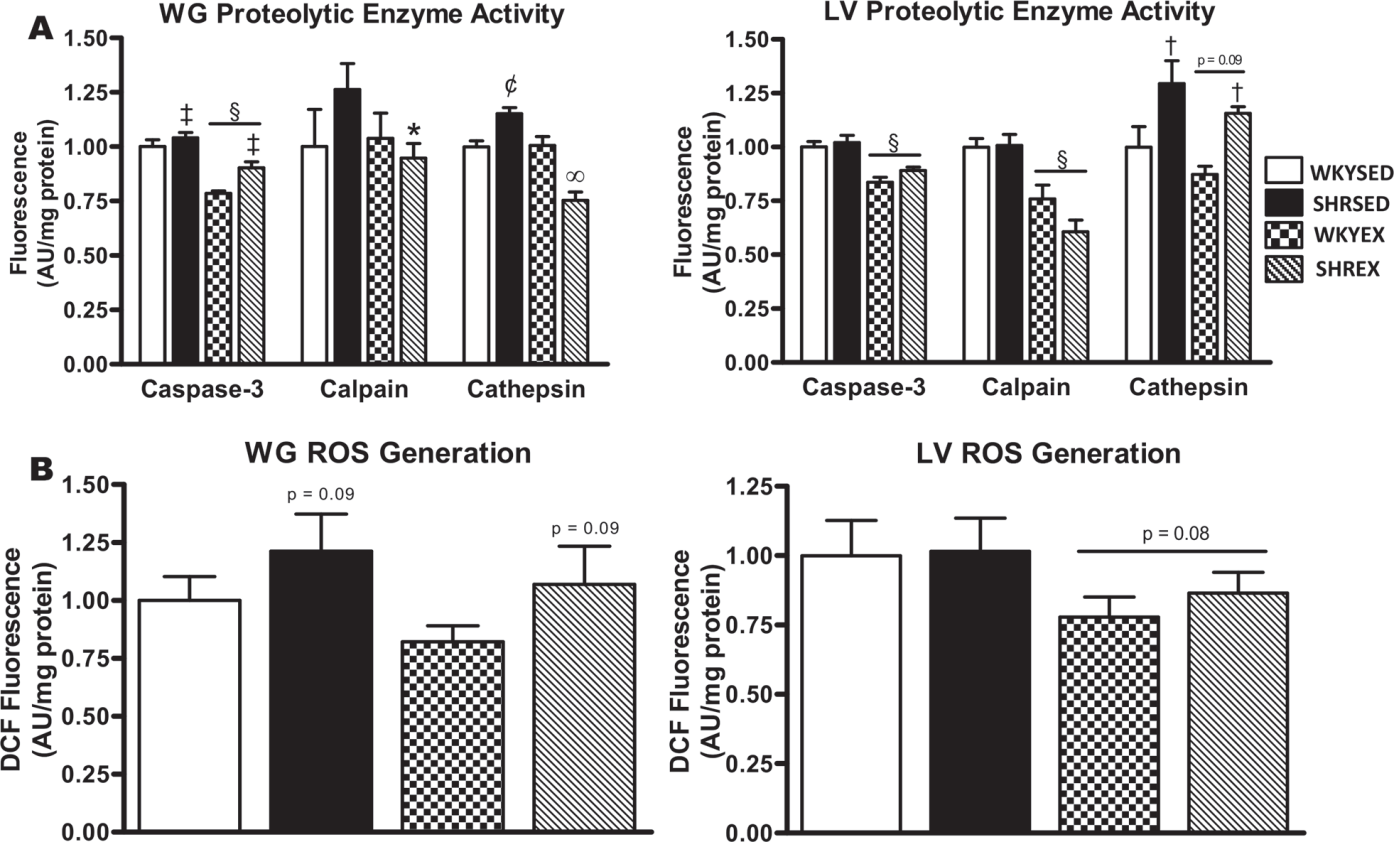
Proteolytic enzyme activity and ROS generation in muscle of sedentary and exercise-trained normotensive and hypertensive rats. *A*: quantitative analysis of caspase-3, calpain, and cathepsin enzymatic activity in the white gastrocnemius (WG) and left ventricle (LV). *B*: quantitative analysis of ROS generation in the WG and LV. Values are means ± SEM (*n* = 9–12). ^†^ p<0.001 vs WKY (main effect); ^‡^ p<0.005 vs WKY (main effect); ^§^ p<0.001 vs SED (main effect); ^¢^ p<0.05 vs all groups (interaction effect); ^∞^ p<0.001 vs all groups (interaction effect); * p<0.05 vs. SHRSED (T-test).

The activity of the apoptosis-related enzyme caspase-3 was higher (p<0.005) in the WG of SHR compared to WKY rats, but was significantly (p<0.001) lower with training. The activity of the lysosomal enzyme cathepsin was elevated (p<0.05) in the WG of the SHRSED group compared to all groups, and significantly (p<0.001) reduced in SHREX animals. Calpain activity was elevated by 26.3% in the SHRSED compared to WKYSED group. T-test analysis revealed that calpain activity was significantly (p<0.05) reduced by 24.9% in exercise-trained compared to sedentary hypertensive animals (SHREX vs. SHRSED) (Fig. 1A). Caspase-3 activity was not significantly different between strains in the LV, but was significantly (p<0.001) lower with training. In the LV, cathepsin activity was elevated (p<0.001) in SHR compared to WKY animals, with exercise training tending (p = 0.09) to reduce cathepsin activity. Calpain activity was not different across strains in the LV; however, exercise training reduced (p<0.001) LV calpain activity (Fig. 1A).

ROS generation tended (p = 0.09) to be greater in the WG of SHR compared to WKY rats; however, exercise did not alter WG ROS generation. In the LV, no difference was observed in ROS generation between SHR and WKY animals (Fig. 1B). However, exercise training tended to reduce (p = 0.08) LV ROS generation.

**Fig 2:**
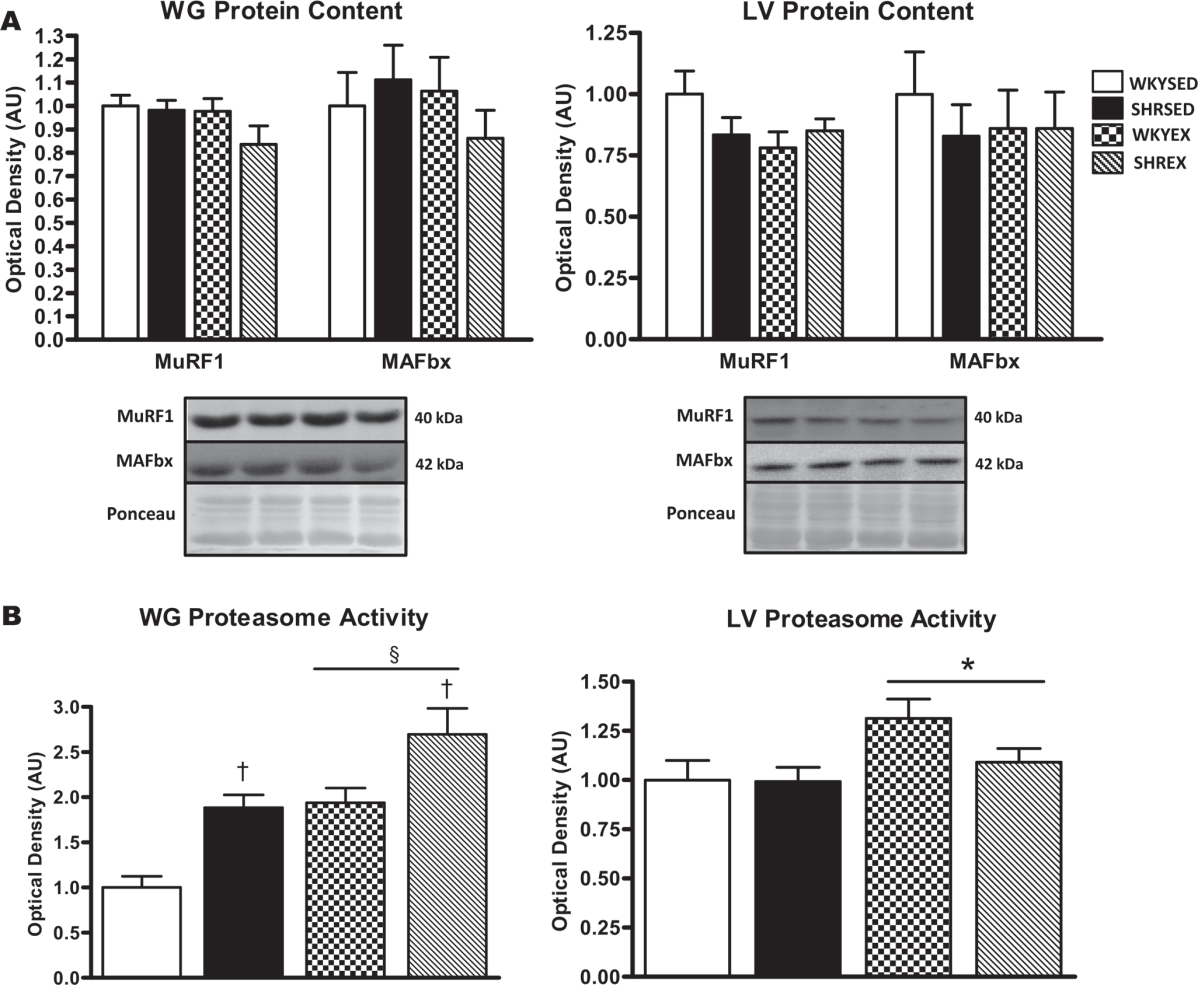
Ubiquitin-proteasome system (UPS)-related proteins and activity in muscle of sedentary and exercise-trained normotensive and hypertensive rats. *A*: quantitative analysis and representative immunoblots of MuRF1 and MAFbx protein in WG and LV. Portions of Ponceau stained membranes are also shown to verify equal loading and quality of transfer. *B*: quantitative analysis of proteasome activity in the WG and LV. Values are means ± SEM (*n* = 9–12). ^†^ p<0.001 vs WKY (main effect); * p<0.05 vs SED (main effect); ^§^ p<0.001 vs SED (main effect).

MAFbx and MuRF1 protein content did not differ between strains and was not affected by exercise in both the WG and LV (Fig. 2A). However, proteasome activity in the WG was significantly higher (p<0.001) in SHR compared to WKY animals, and further elevated (p<0.001) with exercise (Fig. 2B). Proteasome activity was also elevated (p<0.05) with exercise training in the LV (Fig. 2B).

### Expression of mRNA and proteins related to autophagosome content and formation

**Fig 3:**
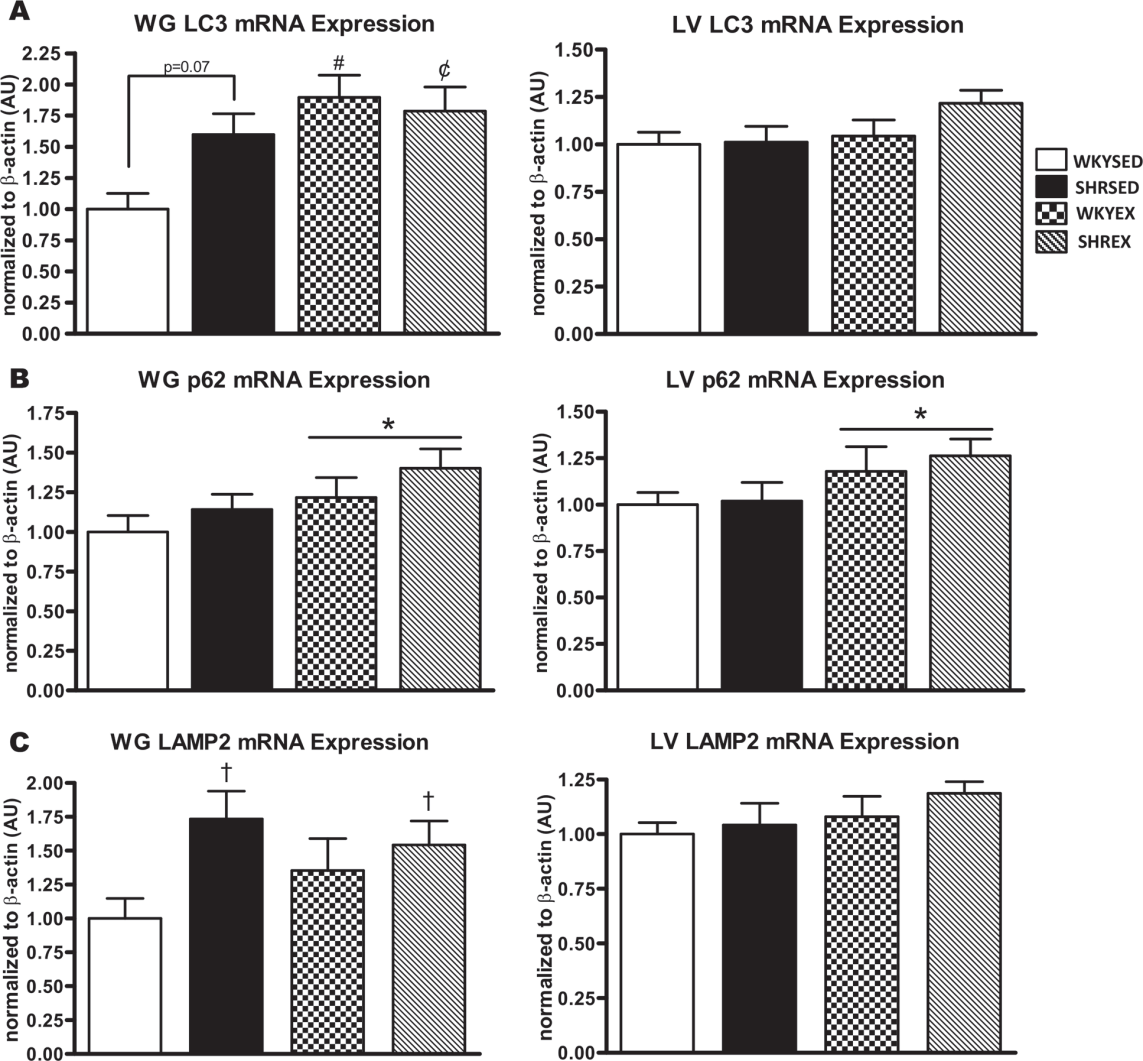
Autophagy-related mRNA expression in muscle of sedentary and exercise-trained normotensive and hypertensive rats. *A*: quantitative analysis of LC3 mRNA in WG and LV. *B*: quantitative analysis of p62 mRNA in WG and LV. *C*: quantitative analysis of LAMP2 mRNA in WG and LV. Values are means ± SEM (*n* = 10–12). ^†^ p<0.05 vs WKY (main effect); * p<0.05 vs SED (main effect); ^¢^ p<0.05 vs WKYSED (interaction effect); ^#^ p<0.005 vs WKYSED (interaction effect).

**Fig 4:**
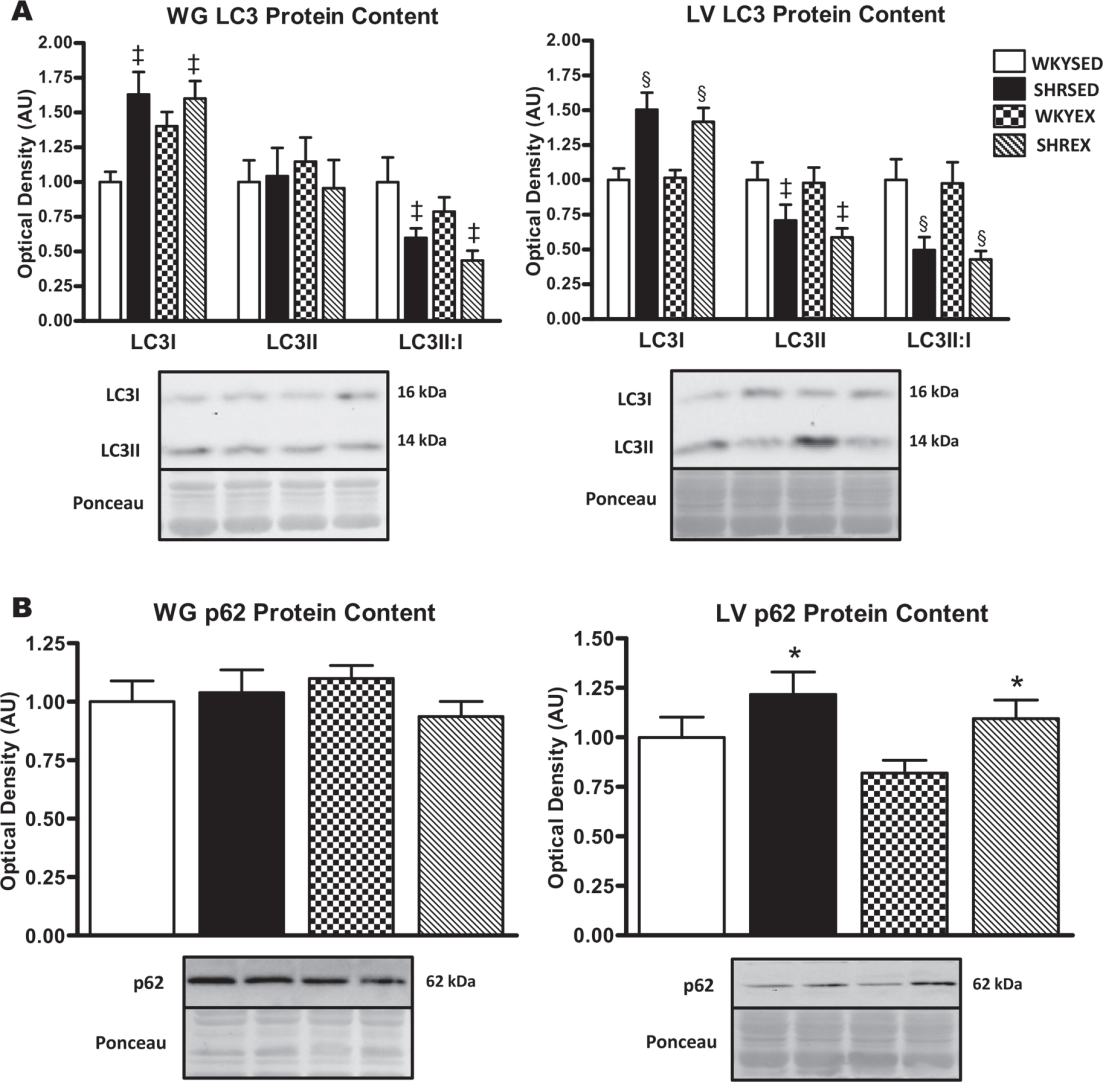
Autophagosome-associated protein expression in muscle of sedentary and exercise-trained normotensive and hypertensive rats. *A*: quantitative analysis and representative immunoblots of LC3 protein in WG and LV (calculated LC3II:I ratio is also shown). *B*: quantitative analysis and representative immunoblots of p62 protein in WG and LV. Portions of Ponceau stained membranes are also shown to verify equal loading and quality of transfer. Values are means ± SEM (*n* = 11–12). * p<0.05 vs WKY (main effect); ^‡^ p<0.005 vs. WKY (main effect); ^§^ p<0.001 vs WKY (main effect).

To examine if the observed changes in proteolytic markers were related to autophagy, we measured several key factors indicative of autophagosome content. There was a trend (p = 0.07) for elevated LC3 mRNA in the WG of SHRSED compared to the WKYSED group. In addition, LC3 mRNA was significantly higher in the WKYEX (p<0.005) and SHREX (p<0.05) groups compared to the WKYSED group (Fig. 3A). p62 mRNA in the WG was not different across strains but was significantly (p<0.05) higher with exercise training (Fig. 3B). Higher levels of LAMP2 mRNA (p<0.05) were present in hypertensive WG muscle, but were not affected by exercise (Fig. 3C). In the WG, a significant increase (p<0.005) in LC3I protein was found in hypertensive animals, but there was no change with exercise. LC3II protein did not differ in the WG across strains or by training status. The LC3II:I ratio was lower (p<0.005) in the WG of hypertensive rats, but was not affected by exercise (Fig. 4A).

In the LV, LC3 and LAMP2 mRNA levels were not different across strains or exercise condition (Fig. 3A & C). p62 mRNA levels were not different across strains in the LV, but were significantly (p<0.05) higher with exercise (Fig. 3B). In the LV, LC3I protein was significantly higher (p<0.001) while LC3II protein was lower (p<0.005) in hypertensive rats, resulting in a lower (p<0.001) LC3II:I ratio. Exercise training did not alter LC3I or LC3II protein, as well as the LC3II:I ratio in the LV (Fig. 4A). In the WG, p62 protein was not significantly different across groups and did not change with exercise training (Fig. 4B). In the LV, p62 protein was higher (p<0.05) in SHR compared to WKY rats, but was not altered with exercise (Fig. 4B).

**Fig 5:**
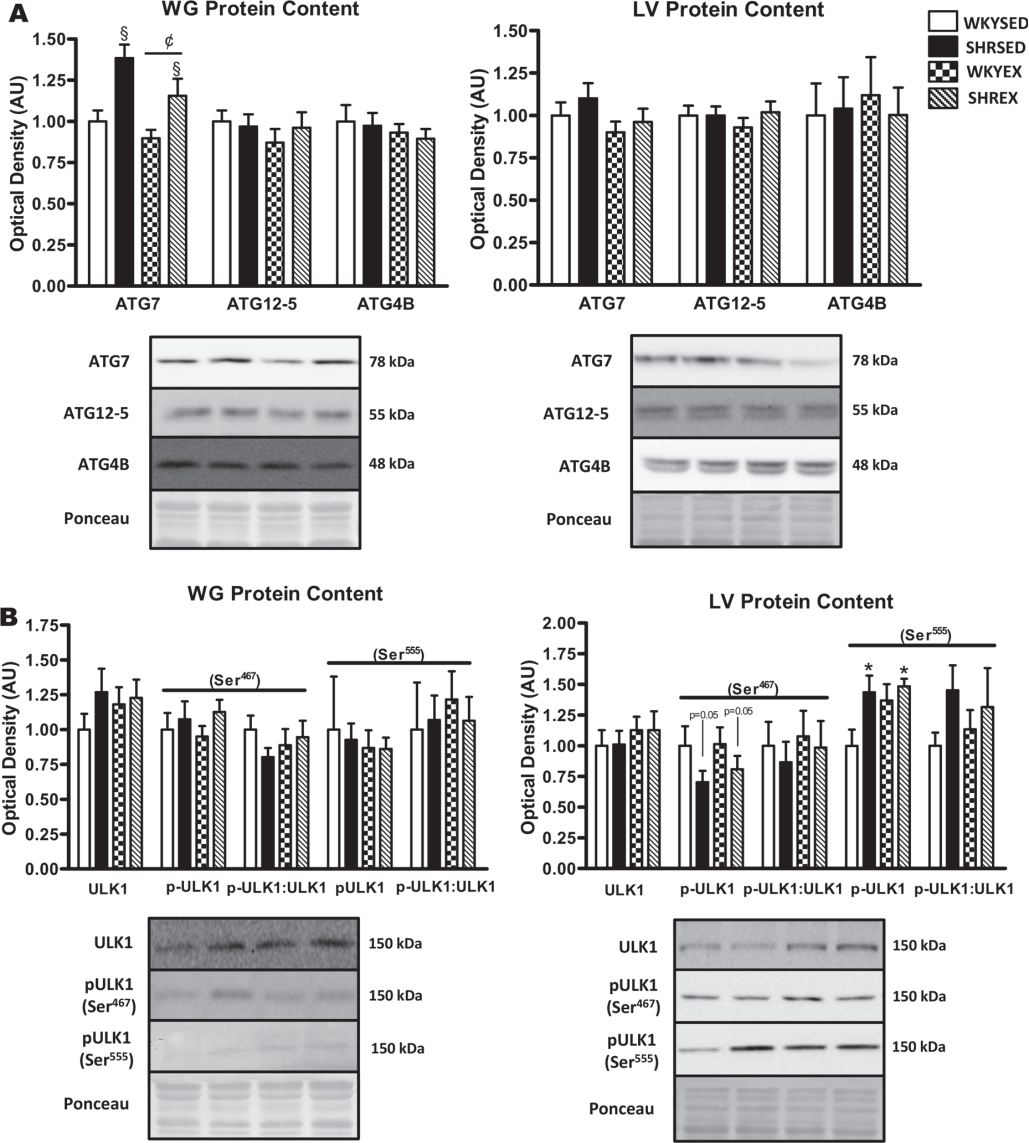
Expression of autophagic proteins in muscle of sedentary and exercise-trained normotensive and hypertensive rats. *A*: representative immunoblots and quantitative analysis of ATG7, ATG12–5, and ATG4B protein in WG and LV. *B*: representative immunoblots and quantitative analysis of ULK1, p-ULK1 (Ser^467^), and p-ULK1 (Ser^555^) in WG and LV (calculated p-ULK1:ULK1 ratios are also shown). Portions of Ponceau stained membranes are also shown to verify equal loading and quality of transfer. Values are means ± SEM (*n* = 5–12). * p<0.05 vs WKY (main effect). ^§^ p<0.001 vs WKY (main effect); ^¢^ p<0.05 vs SED (main effect).

In the WG, ATG7 protein was significantly greater (p<0.001) in hypertensive animals, and reduced (p<0.05) with exercise (Fig. 5A). No differences in ATG4B or ATG12-5 protein were found in the WG between strains or training groups (Fig. 5A). In the LV, no differences in ATG7, ATG4B, or ATG12–5 protein were found across strains or training groups (Fig. 5A).

### Expression of mRNA and proteins involved in autophagy regulation and induction

To examine autophagy induction pathways we measured ULK1 protein content and phosphorylation. In the WG, total ULK1 protein, p-ULK1 (Ser^467^), p-ULK1 (Ser^555^), the p-ULK1 (Ser^467^):ULK1 ratio, and the p-ULK1 (Ser^555^):ULK1 ratio were not different across strains or exercise groups (Fig. 5B). In the LV, total ULK1 protein, the p-ULK1 (Ser^467^):ULK1 ratio, and the p-ULK1 (Ser^555^):ULK1 ratio were not different across strains or exercise groups. However, p-ULK1 (Ser^467^) was lower (p = 0.05) while p-ULK1 (Ser^555^) was higher (p<0.05) in the LV of hypertensive rats, but were not affected by exercise (Fig. 5B).

**Fig 6:**
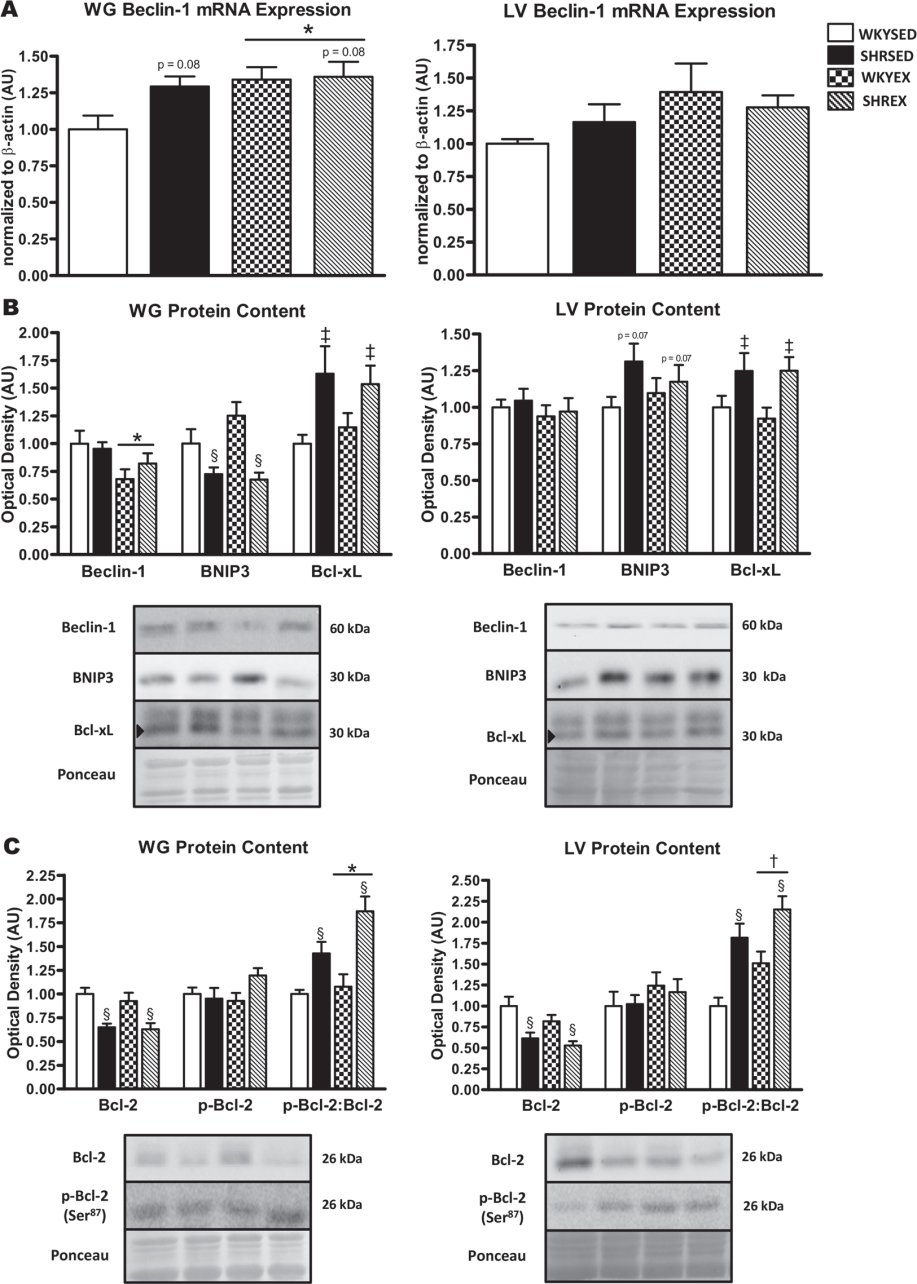
Expression of mRNA and proteins involved in autophagy regulation and induction in muscle of sedentary and exercise-trained normotensive and hypertensive rats. *A*: quantitative analysis of Beclin-1 mRNA in WG and LV. *B*: representative immunoblots and quantitative analysis of Beclin-1, BNIP3, and Bcl-xL protein in WG and LV. *C*: representative immunoblots and quantitative analysis of Bcl-2 and p-Bcl-2 (Ser^87^) protein in WG and LV (calculated p-Bcl-2:Bcl-2 ratio is also shown). Portions of Ponceau stained membranes are also shown to verify equal loading and quality of transfer. Values are means ± SEM (*n* = 9–12). ^‡^ p<0.005 vs WKY (main effect); ^§^ p<0.001 vs WKY (main effect); * p<0.05 vs SED (main effect); ^†^ p<0.01 vs SED (main effect).

An additional regulatory pathway of autophagy induction is through the Bcl-2/Beclin-1 complex. In the WG, Beclin-1 mRNA tended (p = 0.08) to be higher in hypertensive animals and was higher (p<0.05) with exercise training (Fig. 6A). Beclin-1 protein in the WG did not differ between strains, but was reduced (p<0.05) with exercise (Fig. 6B). In the LV, Beclin-1 mRNA (Fig. 6A) and Beclin-1 protein (Fig. 6B) did not differ between strains or with exercise.

Beclin-1 activity is regulated by apoptosis-related proteins such as those of the Bcl-2 family. In the WG, Bcl-2 protein was significantly (p<0.001) lower in SHR compared to WKY rats, but was not altered with exercise (Fig. 6C). Phosphorylation of Bcl-2 at serine 87 dissociates this anti-apoptotic protein from the Beclin-1 complex resulting in increased Beclin-1 activity and the promotion of autophagy [26]. In the WG, p-Bcl-2 (Ser^87^) did not differ across strains or with exercise. However, the p-Bcl-2:Bcl-2 ratio was higher (p<0.001) in the WG of hypertensive animals, and further elevated (p<0.05) with training (Fig. 6C). Bcl-xL protein in the WG was significantly (p<0.005) higher in SHR compared to WKY rats, but was not affected by exercise (Fig. 6B). BNIP3 is a pro-autophagic protein that can promote autophagy through both Beclin-1-dependent and-independent means [27]. In the WG, BNIP3 protein was lower (p<0.001) in hypertensive animals, but not altered by exercise (Fig. 6B).

In the LV, Bcl-2 protein was lower (p<0.001) in SHR compared to WKY animals, but was not affected by exercise (Fig. 6C). p-Bcl-2 (Ser^87^) in the LV did not differ across strains or with exercise. However, the p-Bcl-2:Bcl-2 ratio was higher (p<0.001) in the LV of hypertensive animals, and further elevated (p<0.01) with training (Fig. 6C). Bcl-xL protein in the LV was significantly (p<0.005) higher in SHR compared to WKY rats, but not altered by exercise (Fig. 6B). A trend (p = 0.07) towards greater BNIP3 protein expression was found in the LV of hypertensive rats; however, BNIP3 protein was not altered by exercise (Fig. 6B).

### Protein expression and phosphorylation status of AKT, AMPK, FoxO3a, and p70S6K

**Fig 7:**
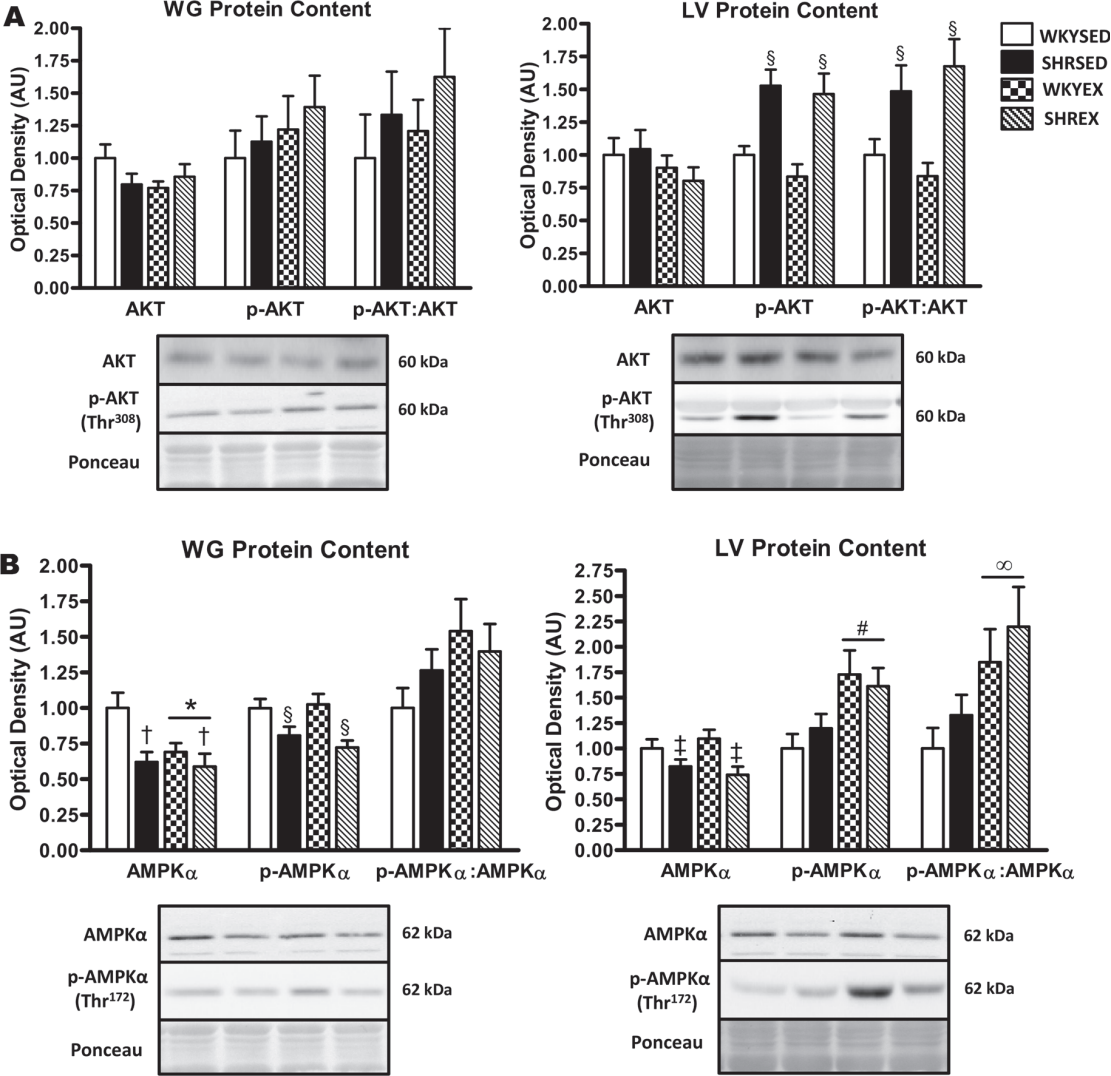
Expression and phosphorylation status of AKT and AMPKα protein in muscle of sedentary and exercise-trained normotensive and hypertensive rats. *A*: quantitative analysis and representative immunoblots of AKT and p-AKT (Thr^308^) protein in WG and LV (calculated p-AKT:AKT ratio is also shown). *B*: quantitative analysis and representative immunoblots of AMPKα and p-AMPKα (Thr^172^) protein in WG and LV (calculated p-AMPKα:AMPKα ratio is also shown). Portions of Ponceau stained membranes are also shown to verify equal loading and quality of transfer. Values are means ± SEM (*n* = 8–12). ^†^ p<0.01 vs WKY (main effect); ^‡^ p<0.005 vs WKY (main effect); ^§^ p<0.001 vs WKY (main effect); * p<0.05 vs SED (main effect); ^∞^ p<0.01 vs SED (main effect); ^#^ p<0.005 vs SED (main effect).

To further investigate autophagic signaling, we examined several upstream kinases and downstream targets. In the WG, there was no difference in total AKT protein, p-AKT (Thr^308^), or the p-AKT:AKT ratio between strains or training groups (Fig. 7A). Total AKT protein was not different by strain or exercise in the LV. p-AKT (Thr^308^) and the p-AKT:AKT ratio were higher in the LV (p<0.001) of hypertensive animals, but were not affected by exercise (Fig. 7A). AMPK can promote autophagic signaling through ULK [28]. In the WG, hypertensive animals had lower AMPKα (p<0.01) and p-AMPKα (Thr^172^) (p<0.001) protein expression, but there was no difference in the p-AMPKα:AMPKα ratio. Furthermore, AMPKα protein (p<0.05) was lower while p-AMPKα (Thr^172^) levels and the p-AMPKα:AMPKα ratio were not altered with exercise in the WG (Fig. 7B). In the LV, hypertensive animals had lower AMPKα protein (p<0.005) but showed no change in p-AMPKα (Thr^172^) or the p-AMPKα:AMPKα ratio. However, p-AMPKα (Thr^172^) levels (p<0.005) and the p-AMPKα:AMPKα ratio (p<0.01) were higher in LV of exercised animals (Fig. 7B).

**Fig 8:**
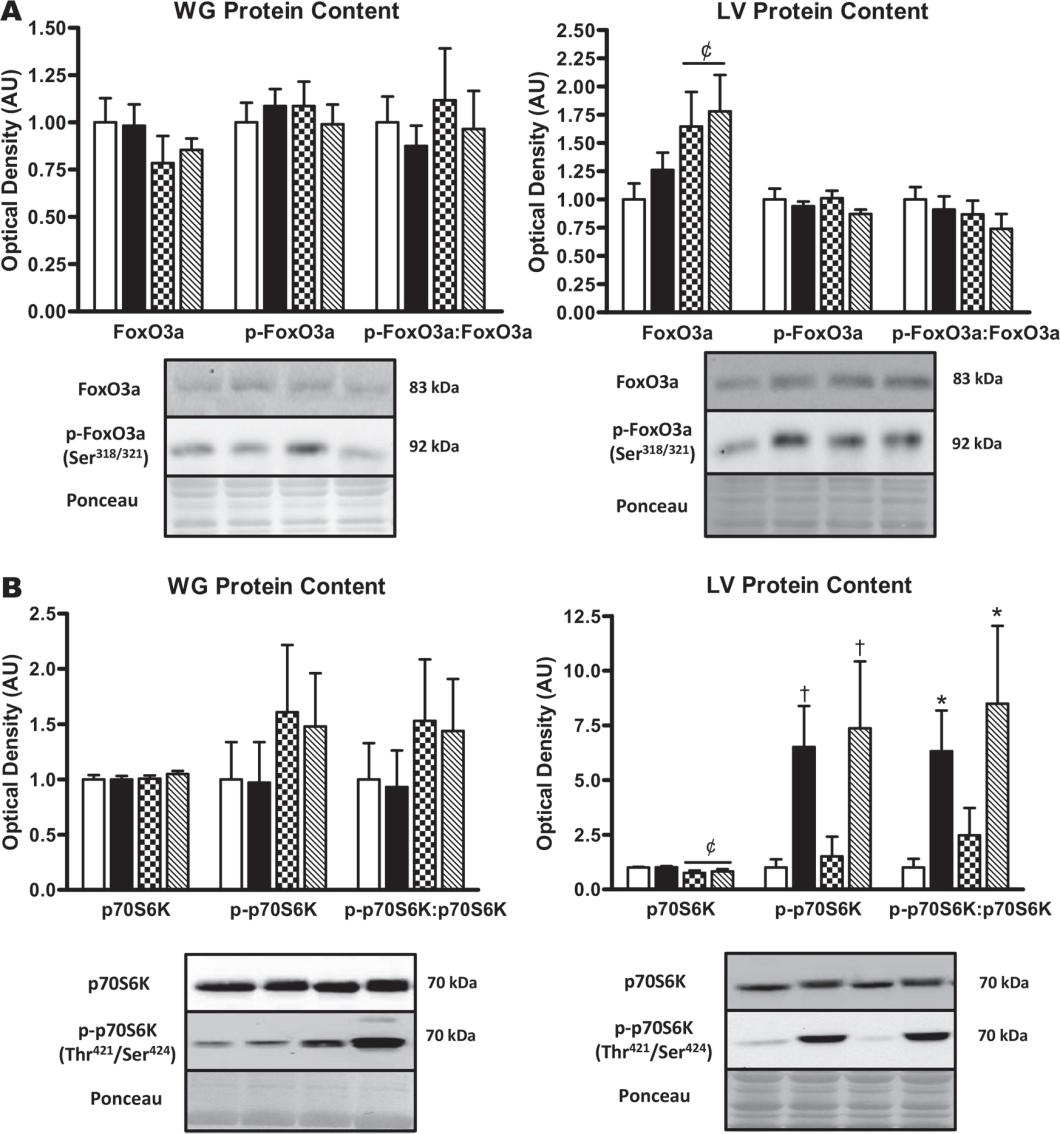
Expression and phosphorylation status of FoxO3a and p70S6K protein in muscle of sedentary and exercise-trained normotensive and hypertensive rats. *A*: quantitative analysis and representative immunoblots of FoxO3a and p-FoxO3a (Ser^318/321^) protein in WG and LV (calculated p-FoxO3a:FoxO3a ratio is also shown). *B*: quantitative analysis and representative immunoblots of p70S6K and p-p70S6K (Thr^421^/Ser^424^) protein in WG and LV (calculated p-p70S6K:p70S6K ratio is also shown). Portions of Ponceau stained membranes are also shown to verify equal loading and quality of transfer. Values are means ± SEM (*n* = 4–12). * p<0.05 vs WKY (main effect); ^†^ p<0.01 vs WKY (main effect); ^¢^ p<0.05 vs SED (main effect).

We next examined FoxO3a content as it is a major transcription factor for a number of autophagy genes downstream of AKT [29]. There were no differences by strain or exercise status in total FoxO3a protein, p-FoxO3a (Ser^318/321^), or the p-FoxO3a:FoxO3a ratio in the WG (Fig. 8A). In the LV, no difference in total FoxO3a protein was found across strains; however, FoxO3a protein was higher (p<0.05) with exercise. There were no differences in p-FoxO3a (Ser^318/321^) levels or the p-FoxO3a:FoxO3a ratio in the LV by strain or exercise condition (Fig. 8A). We also examined the levels of p70S6K, a major target of mTOR [30]. There were no differences in p70S6K protein, p-p70S6K (Thr^421^/Ser^424^), or the p-p70S6K:p70S6K ratio in the WG across strains or exercise groups (Fig. 8B). In the LV, no difference in total p70S6K protein was found between strains; however, p70S6K protein was lower (p<0.05) with exercise. In addition, p-p70S6K (Thr^421^/Ser^424^) levels (p<0.01) and the p-p70S6K:p70S6K ratio (p<0.05) were dramatically elevated in LV of hypertensive rats, but not affected by exercise (Fig. 8B).

## Discussion

We have previously demonstrated that apoptosis and autophagy are upregulated in the skeletal muscle of hypertensive rats [18–21]. In addition, we have previously shown that exercise training can decrease apoptotic signaling in skeletal muscle [20]. In this investigation, we found that exercise training alters the proteolytic environment in skeletal and cardiac muscle of hypertensive animals, and influences several factors involved in autophagy in skeletal muscle.

### Exercise training reduces apoptosis-, calpain-, and lysosome-related enzyme activity but enhances proteasome activity in skeletal muscle

Myopathies are often associated with alterations to apoptotic processes, the ubiquitin-proteasome system (UPS), and the autophagy-lysosome system [31–33]. Consistent with our previous reports, we found that skeletal muscle of hypertensive rats had significantly greater activity of the apoptosis-related enzyme caspase-3 [18,20], and lysosomal enzyme cathepsin [21], as well as higher ROS generation [19,20]. In addition, enzymatic activity of calpains and the proteasome were higher in the skeletal muscle of hypertensive rats. Collectively, these data suggest that degradative enzymes and pathways are elevated in skeletal muscle of hypertensive animals. Interestingly, chronic exercise training was able to reduce skeletal muscle caspase-3, calpain, and cathepsin activity, which is in agreement with previous reports [34–36]. In contrast, exercise training caused a further increase in proteasome activity. This is consistent with a recent report, which found that proteasome activity was increased in the plantaris muscle of mice following 8 weeks of aerobic exercise training [25]. The lower caspase-3 and calpain proteolytic activity with exercise is likely due to decreased upstream activation of apoptotic pathways [20,34], as well as possible alterations to autophagy. Further, augmented UPS-mediated clearance of damaged/dysfunctional proteins through increased proteasome activity may account for the decreased apoptotic signaling. Elevated caspase-3 activation is observed in muscle of aged MuRF1 knockout mice [37] and in C2C12 cells treated with a proteasome inhibitor [38], supporting a critical role of the UPS in regulating apoptotic signaling. Together, our data support the role of exercise training as a means of mitigating the apoptotic and lysosomal proteolytic environment in skeletal muscle, possibly through enhanced proteasome activation. Previous work has demonstrated that skeletal muscle of SHR display a number of morphological and functional impairments/alterations [21,39,40]. Interestingly, suppression of calpain and caspase activation has also been shown to reduce muscle atrophy and contractile dysfunction [41,42]. It remains to be determined if the exercise-mediated reductions in calpain, caspase-3, and cathepsin activity observed in this study would correct the functional and morphological alterations observed in SHR skeletal muscle. In support of this notion, a recent report found that exercise-mediated reductions in calpain and caspase-3 activation were associated with reduced muscle proteolysis following doxorubicin administration [36].

### Exercise training alters the expression of autophagic factors in skeletal muscle

Chronic diseases that directly [43,44], or indirectly [21,45] affect skeletal muscle are commonly associated with alterations in autophagy. Consistent with our previous data [21], skeletal muscle of hypertensive animals displays a number of alterations in autophagy-specific markers (i.e., LC3 and LAMP2 mRNA, LC3I protein and LC3II:I ratio, and ATG7 protein), suggesting an increase in autophagic activation and clearance. Chronic exercise training was effective in increasing LC3, p62, and Beclin-1 mRNA, as well as attenuating ATG7 and Beclin-1 protein levels in skeletal muscle. Recent findings show that exercise induces the lipidation of LC3 and the degradation of p62 [12], supporting the role of acute exercise as an activator of autophagy. However, single bouts of exercise have also been found to decrease autophagy-related protein expression in skeletal muscle [13]. Taken together, the lower Beclin-1 and ATG7 protein levels along with the reduced cathepsin activity suggest that chronic exercise training lowers autophagic factors in skeletal muscle. Since muscle was isolated 24 hours following exercise, it is possible that the elevated mRNA expression reflects an acute effect of the treadmill running, whereas lower levels of autophagy-related proteins are indicative of a chronic training adaptation. Further research is required to elucidate these contrasting patterns of expression during chronic exercise training.

### Altered autophagic factors in skeletal muscle with exercise training are not associated with sustained AMPK-ULK, AKT-FoxO3, or AKT-mTOR signaling

AMPK can contribute to autophagy induction in skeletal muscle through direct interactions and phosphorylation of ULK1 [46]. Consistent with previous data [47], we found lower AMPKα and AMPKα phosphorylation in skeletal muscle of hypertensive rats. Despite this, there were no differences in ULK1 or ULK1 phosphorylation. In addition, p-AMPKα levels and the p-AMPKα:AMPKα ratio were not altered with exercise despite a reduction in AMPKα protein. AMPK activation has been shown to increase in skeletal muscle immediately after exercise, but returns to pre-exercise levels within 3 hours [48]. Since our animals were sacrificed 24 hours following exercise, our findings are not surprising. AKT can also regulate autophagy via mTOR and FoxO3a signaling [49]. However, alterations in AKT and FoxO3a signaling were not found in skeletal muscle during hypertension or with exercise. Consistent with the AKT data, p70S6K and p-p70S6K levels (a major target of mTOR) were not different between strains or with exercise training in skeletal muscle. Together, these data suggest that sustained AMPK-ULK, AKT-FoxO3a, and AKT-mTOR signaling are likely not responsible for the altered autophagic factors observed in skeletal muscle during hypertension, and are not a characteristic of chronically trained muscle. It remains to be determined if prior activation of these pathways by the acute aspects of exercise training influence some of the observed alterations.

### Exercise training reduces Beclin-1 and increases the p-Bcl-2:Bcl-2 ratio in skeletal muscle

The formation of Bcl-2:Beclin-1 complexes results in reduced autophagy [6]. In addition, phosphorylation of Beclin-1 and/or Bcl-2 can inhibit the Bcl-2:Beclin-1 interaction and induce autophagy [6,26]. We found significantly lower Bcl-2 protein and a higher p-Bcl-2:Bcl-2 ratio, along with similar Beclin-1 protein in skeletal muscle of hypertensive animals. Thus, these findings further support an increase in autophagic signaling in skeletal muscle of hypertensive animals. While phosphorylation of Bcl-2 at serine 87 would promote autophagy [6], it has also been demonstrated that this could reduce [50,51] or promote [52,53] Bcl-2’s anti-apoptotic action. Furthermore, phosphorylation of Bcl-2 at serine 87 can protect it from degradation and preserve its anti-apoptotic function [54,55]. Thus, the direct role of this altered ratio in hypertensive skeletal muscle remains to be determined in the context of dramatically reduced Bcl-2 levels. Interestingly, Bcl-xL protein was elevated in hypertensive skeletal muscle. Although Bcl-xL can interact with Beclin-1 to mitigate autophagy, upregulation of Bcl-xL is also associated with increased autophagy and cell death signaling [56,57]. Collectively, the present investigation clearly demonstrates sizable alterations in several Bcl-2 family proteins in skeletal muscle during hypertension, which may play a critical role in cell death and degradative processes.

Acute exercise has been shown to cause a dissociation of Bcl-2 from Beclin-1 and the induction of autophagy [12]. We found an elevated p-Bcl-2:Bcl-2 ratio along with reduced Beclin-1 protein in skeletal muscle of trained animals. In this scenario, the lower Beclin-1 content may have abrogated the exercise-induced elevation in the p-Bcl-2:Bcl-2 ratio, thus not significantly influencing autophagy. Alternatively, the elevated p-Bcl-2:Bcl-2 ratio in skeletal muscle following training may have a purely apoptotic role, and would be consistent with the reduced caspase-3 and calpain activity we observed.

### Exercise training alters proteolytic enzyme activity but not autophagic factors in LV

Activation of degradative and proteolytic processes in the heart contributes to pathological tissue remodeling and cardiac dysfunction during conditions such as hypertension [58,59]. Similar to the results obtained in skeletal muscle, exercise training significantly reduced LV caspase-3 and calpain enzymatic activity, with a trend towards reduced cathepsin activity and ROS generation. Consistent with our skeletal muscle data, exercise training enhanced proteasome activity in the LV. Taken together, these data suggest that exercise training can attenuate caspase- and calpain-related signaling in the LV, possibly through enhanced proteasome activation. Whether this exercise-mediated reduction in caspase and calpain activity could impact cardiac function and remodeling during hypertension remains to be determined.

Consistent with our previous report [21], the LV of hypertensive animals displayed elevated LC3I and reduced LC3II (as well as a lower LC3II:I ratio), along with no change in several ubiquitin-like conjugation enzymes (ATG7, ATG4B, and ATG12-5). Importantly, hypertensive LV had elevated p62 protein levels, suggesting a reduction in autophagic signaling, which is further supported by higher AKT and p70S6K phosphorylation (discussed below). Although the consequence of this reduced autophagic signaling is currently unknown, inhibition of autophagy mitigates load-induced hypertrophic remodeling [60]; thus this altered autophagic signaling in SHR heart may be a protective response. Conversely, autophagic inhibition could negatively impact protein and mitochondrial quality control. Although exercise was associated with elevated p62 mRNA in the LV, it did not affect LC3 and LAMP2 mRNA, as well as Beclin-1, ATG12-5, ATG4B, ATG7, LC3I, LC3II, and p62 protein. Previous work has shown increased autophagic protein expression in cardiac muscle with exercise; however, these previous results were obtained immediately following an acute bout of exercise [14]. These discrepancies may be due to the timing of tissue collection as well as differential “acute effects” versus “chronic adaptations” of exercise.

### Exercise training does not alter basal AKT-mTOR, AKT-FoxO3, or AMPK-ULK signaling, but alters the p-Bcl-2:Bcl-2 ratio in LV

Autophagy can be regulated by AKT through FoxO3a translocation and the activation of the mTOR pathway [49], as well as by AMPK through direct phosphorylation of the ULK1 complex [28]. Consistent with previous literature [61], we found elevated p-AKT levels in LV of hypertensive animals; however, FoxO3a and p-FoxO3a protein levels were unchanged, suggesting that this pathway does not directly alter autophagy in this context. However, p70SK6 phosphorylation was dramatically elevated in LV of hypertensive animals suggesting activation of mTOR, and providing further support for a reduced autophagic state in LV during hypertension. Although AMPKα protein was lower in hypertensive heart and would tend to reduce autophagic signaling through ULK [28], we found reduced p-ULK1 (Ser^467^) but elevated p-ULK1 (Ser^555^) levels. Given these opposing responses in ULK phosphorylation, downstream alterations in LV autophagy due to this signaling pathway are unlikely and/or insignificant in the face of high mTOR activation (as suggested by the dramatically elevated p-AKT and p-p70S6K levels). Although AMPKα levels were elevated with exercise in the LV, there was no effect on ULK1 expression or phosphorylation. Furthermore, AKT, p70S6K, and FoxO3a phosphorylation were not altered with exercise. These findings coupled with no change to a number of autophagy markers (i.e., LC3 and LAMP2 mRNA, LC3I, LC3II, Beclin-1, ATG7, and p62 protein, etc) suggest that 6 weeks of chronic exercise training does not significantly affect autophagic signaling in LV in the basal state.

We also found a reduction in anti-apoptotic Bcl-2 protein and an increased p-Bcl-2:Bcl-2 ratio in the LV of hypertensive animals. Bcl-2 can interact with Beclin-1 and participate in autophagic signaling [62]; however, it also has a very well-known inhibitory role in apoptosis [63]. The phosphorylation of Bcl-2 may be an attempt to upregulate autophagy [26]. However, given the lack of change to downstream autophagy-related proteins in the LV, it is unlikely that this effect is playing an autophagic role. Although no differences in caspase-3 were observed in LV between normotensive and hypertensive animals, the reduction in Bcl-2 suggests an increase in apoptotic sensitivity, which is associated with the induction of myocardial impairment during hypertension [64]. Moreover, the increase in the p-Bcl-2:Bcl-2 ratio in hypertension may also be indicative of altered Bcl-2 function (as discussed above). The p-Bcl-2:Bcl-2 ratio was further altered with exercise training in the LV; however, no concomitant changes to downstream effectors of autophagy were observed. Although this altered p-Bcl-2:Bcl-2 ratio may have a potential apoptotic role, its precise role remains to be determined in this context.

## Conclusion

As a whole, our data show altered autophagic signaling in cardiac and skeletal muscle of hypertensive animals. In addition, we demonstrate that regular exercise training can alter proteolytic enzyme activity in both cardiac and skeletal muscle, as well as influence the expression of several autophagy-related factors in skeletal muscle. Overall, these data suggest that regular exercise may be an effective approach for modulating multiple degradative processes in skeletal and cardiac muscle. Future research should work towards elucidating if the observed “chronic training” adaptations are due to repeated “acute-exercise” induction of autophagy and subsequent removal of harmful factors, thereby improving the skeletal muscle environment and reducing the need for autophagy in the basal state. In addition, given that there is evidence of differential autophagic and apoptotic signaling across muscles, future work should investigate the influence of exercise on these processes in muscles with different fiber types and metabolic properties.
